# Long-term survival after definitive proton beam therapy for oligorecurrent esophageal squamous cell carcinoma: a case report

**DOI:** 10.1186/s13256-022-03275-0

**Published:** 2022-02-14

**Authors:** Yojiro Ishikawa, Motohisa Suzuki, Hisashi Yamaguchi, Ichiro Seto, Masanori Machida, Yoshiaki Takagawa, Keiichi Jingu, Yasuyuki Kikuchi, Masao Murakami

**Affiliations:** 1 Department of Radiation Oncology, Southern Tohoku Proton Therapy Center, 7-172, Yatsuyamada, Koriyama, Fukushima 963-8052 Japan; 2 Department of Radiation Oncology, Tohoku University Graduate School of Medicine, 1-1 Seiryo-chou, Aoba-ku, Sendai, 980-8574 Japan

**Keywords:** Esophageal cancer, Proton beam therapy, Liver metastasis, Lymph node metastasis

## Abstract

**Background:**

Radical esophagectomy for esophageal squamous cell carcinoma has improved survival, but the rate of recurrence is high. Patients of recurrent esophageal squamous cell carcinoma after failure of chemotherapy have a poor prognosis. We herein report the achievement of long-term survival after definitive proton beam therapy for oligorecurrent esophageal squamous cell carcinoma after failure of chemotherapy.

**Case presentation:**

A 60-year-old Japanese man was diagnosed as having squamous cell carcinoma of the lower thoracic esophagus (cT2N0M0, stage IIA). He underwent two courses of neoadjuvant chemotherapy with cisplatin and 5-fluorouracil, and esophagectomy with three-field lymphadenectomy was performed. Microscopic findings after resection showed two lymph node metastases (ypT2N1M0, stage IIB). Five months after resection, a computed tomography scan revealed a solitary liver metastasis in the S4 area. He underwent three courses of chemotherapy with cisplatin and 5-fluorouracil; however, positron emission tomography revealed two lymph node metastases. Surgeons recommended second-line chemotherapy, but the patient refused chemotherapy and requested proton beam therapy. We performed proton beam therapy without chemotherapy for the liver metastasis and lymph node metastases, with total doses of 79.2 and 60 Gy relative biological effectiveness, respectively, according to the tumor location. An acute side effect of grade 1 dermatitis occurred after proton beam therapy, but there was no acute or late complication of more than grade 2. The patient remains in complete remission 5 years after treatment without surgery or chemotherapy.

**Discussion and conclusions:**

Proton beam therapy exerted a curative effect on oligorecurrent esophageal squamous cell carcinoma. This is the first report on the achievement of long-term survival after definitive proton beam therapy for oligorecurrent esophageal squamous cell carcinoma.

## Background

Esophageal squamous cell carcinoma (ESCC) is one of the most difficult malignancies to cure because of its early metastasis. Radical esophagectomy has improved the survival of patients with ESCC, but the rate of recurrence is high (27–52%) [1–4]. Cisplatin (CDDP) and 5-fluorouracil (5-FU) are used in first-line chemotherapy for recurrent esophageal cancer after radical esophagectomy; however, patients have a poor prognosis after failure of first-line treatment [5].

Proton beam therapy (PBT) is effective because protons have excellent dose localization according to the Bragg peak compared with photons, and are biologically equivalent to conventional X-ray treatment for cancer [6–9]. We herein report the achievement of long-term survival after proton therapy in an ESCC patient in whom the first-line chemotherapy for postoperative solitary liver metastasis and two lymph node metastases failed. This report is an interesting case report on “off-label” management of a single noncolorectal nonneuroendocrine liver metastasis with lymph nodal disease progression in the paraaortic area.

## Case presentation

One year before presentation to our hospital, a male Japanese patient was diagnosed as having squamous cell carcinoma (SCC) of the lower thoracic esophagus (cT2N0M0, stage IIA) at 60 years of age. He underwent two courses of neoadjuvant chemotherapy with CDDP in addition to 5-FU, and esophagectomy with three-field lymphadenectomy followed by gastric tube reconstruction for SCC of the lower thoracic esophagus was performed. Microscopic findings after resection showed that the tumor was moderately differentiated SCC. Additionally, two lymph node metastases were observed (ypT2N1M0, stage IIB).

**Fig. 1:**
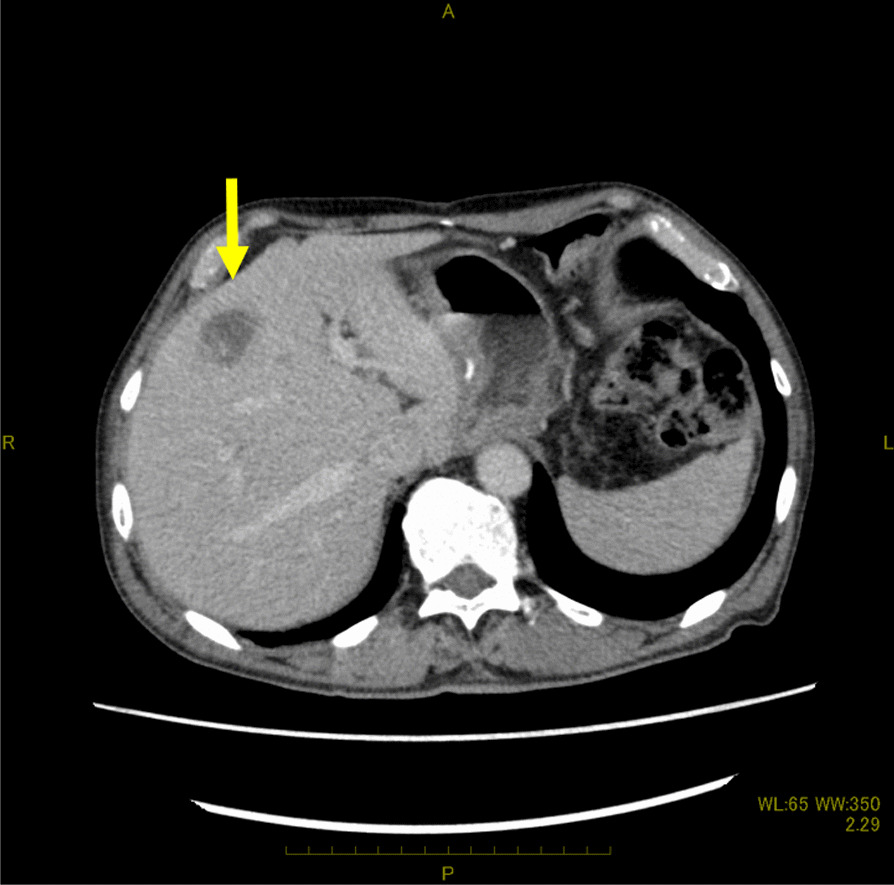
Axial enhanced computed tomography scan images of the abdomen showing a low-enhanced lesion of 21 × 15 mm in size in the S4 area (yellow arrow)

**Fig. 2:**
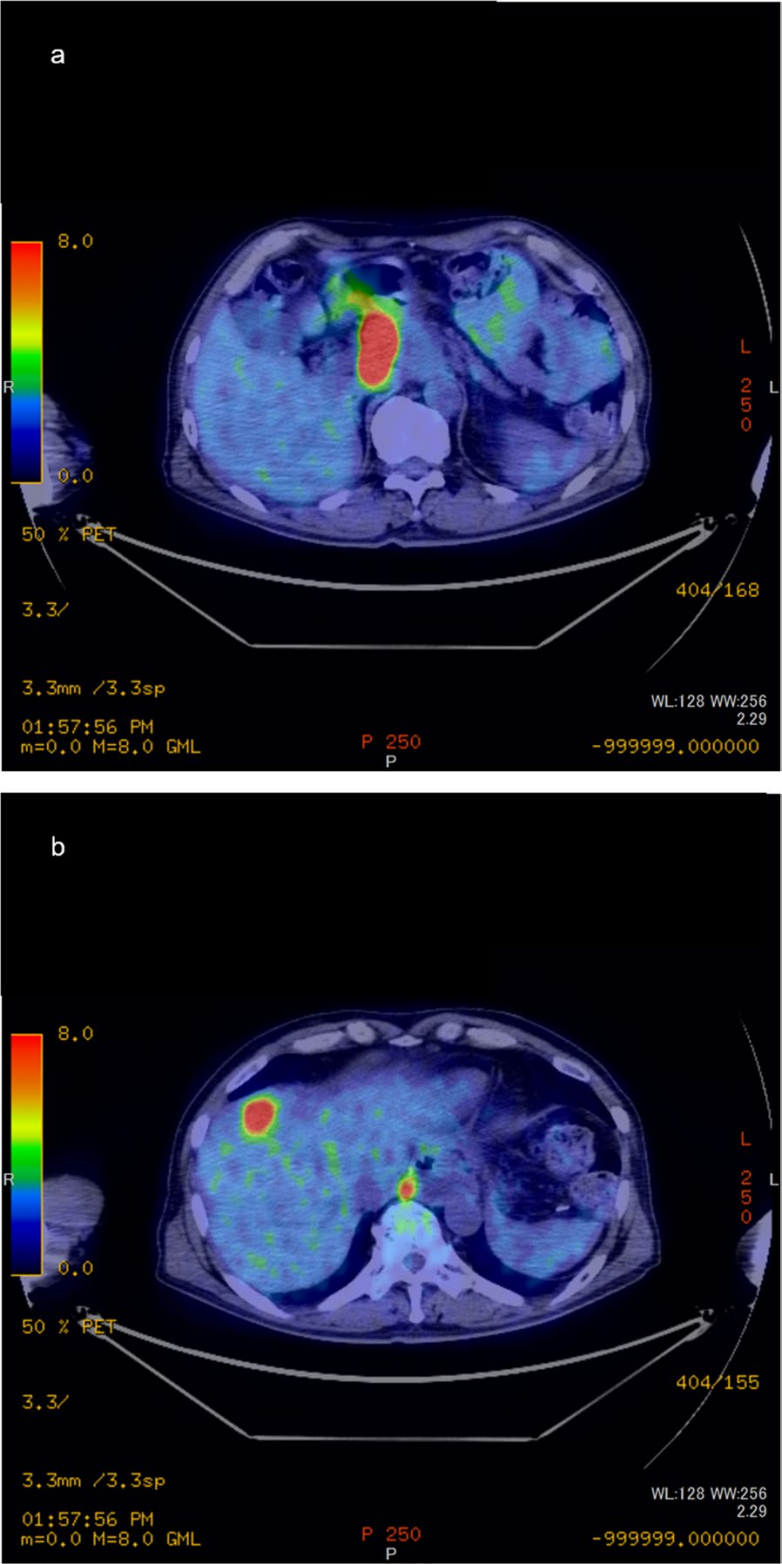
Positron emission tomography before proton beam therapy. Accumulation of fluorodeoxyglucose is shown in (**a**) paraaortic lymph node metastasis and (**b**) liver metastasis in the S4 area with paraaortic lymph node metastasis

Five months after surgery, a computed tomography (CT) scan revealed a solitary liver metastasis measuring 21 × 15 mm in size in the S4 area (Fig. 1). The surgeons did not judge that the preoperative chemotherapy with CDDP in addition to 5-FU had failed. The patient underwent three courses of chemotherapy with CDDP in addition to 5-FU. During the three courses of chemotherapy, the patient suffered from side effects of chemotherapy including nausea and loss of appetite. Follow-up positron emission tomography PET after the three courses of chemotherapy reveled two new lymph node metastases in the paraaortic area (Fig. 2). The surgeons judged that the first-line chemotherapy had failed because the lymph node metastases showed an increase in size.

The surgeons therefore recommended resection of the liver metastasis with retroperitoneal lymph node dissection. However, the patient refused to receive surgery because of concerns about the treatment outcome and side effects, despite the fact that the patient had an Eastern Cooperative Oncology Group Performance Score of 0. The surgeons also recommended chemoradiotherapy or second-line chemotherapy. However, the patient refused to undergo second-line chemotherapy or chemoradiotherapy because of concerns about the treatment side effects.

He requested alternative treatment and was referred to our institution. We considered stereotactic radiotherapy or radio-frequency ablation for the liver metastasis. However, we did not select these therapies because he had two lymph node metastases outside the liver.

We therefore recommended the combination of PBT [or radiation therapy (RT)] and chemotherapy. The patient did not accept the RT and chemotherapy, and he requested PBT without chemotherapy.

We explained the risks and benefits of PBT alone to the patient. One problem with PBT is the high cost of treatment, but the patient was covered by personal medical insurance for PBT. Finally, we decided to perform PBT alone for the liver metastasis and lymph node metastases.

The PBT system at our institute (Proton beam system, Mitsubishi, Tokyo, Japan) uses synchrotron and scattering methods. Treatment planning for PBT was based on three-dimensional CT images taken at 2-mm intervals in the exhalation phase while using a respiratory gating system (Anzai Medical, Tokyo, Japan). Treatment was administered during the exhalation phase using a respiratory gating system. Daily front and lateral X-ray imaging was used for positioning.

The gross tumor volume (GTV) included the liver and lymph node metastases. The clinical target volume (CTV) was defined as GTV plus 0.5-cm margins. The planning target volume (PTV) was CTV plus 0.5-cm margins.

**Fig. 3:**
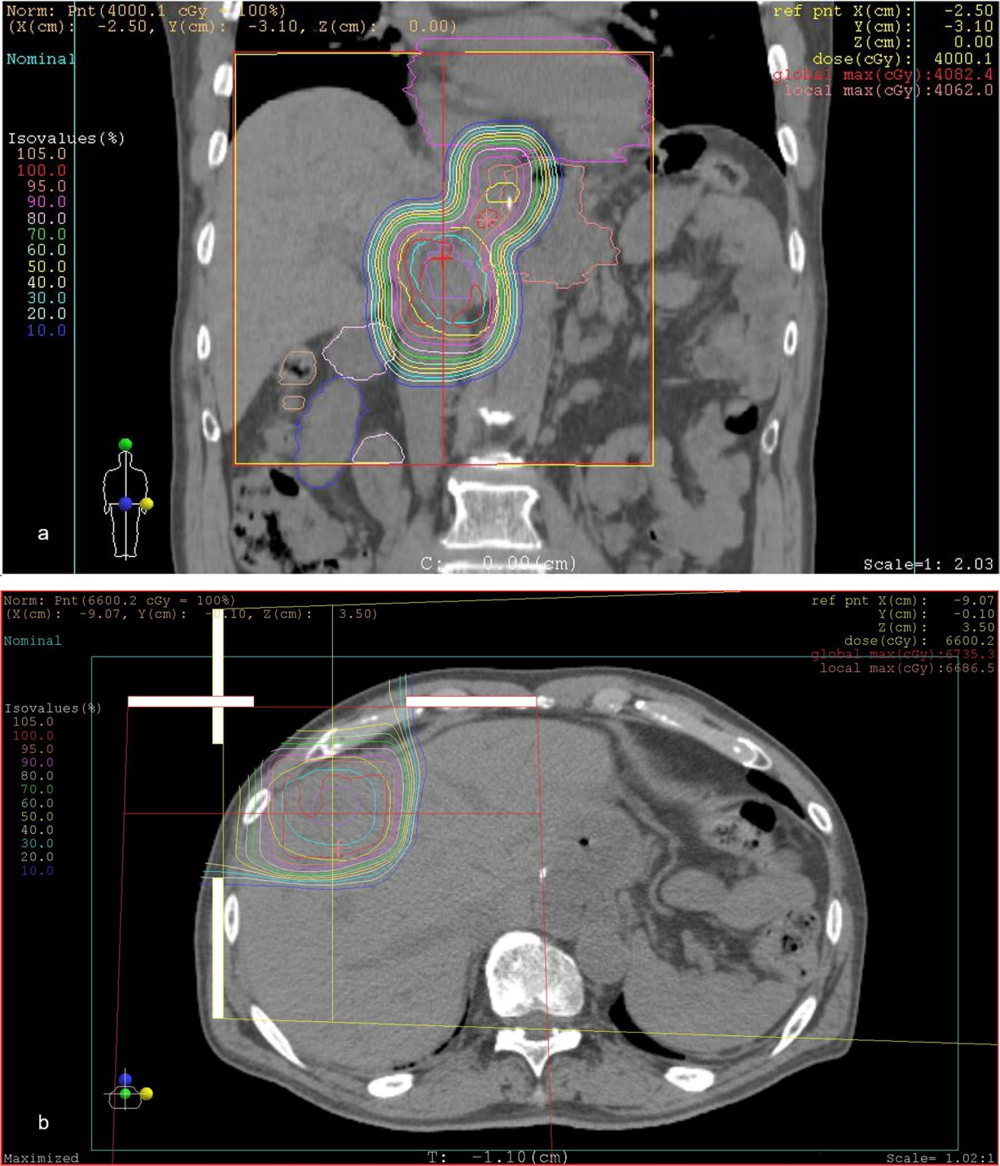
Dose distribution of proton beam therapy. **a** The lymph nodes were treated with 60 Gy relative biological effectiveness. The liver metastasis in S4 (**b**) was treated with 79.2 Gy relative biological effectiveness

We decided to reduce the daily dose for the lymph node metastases because they were located close to the stomach. The daily PBT fractions were 3.3 and 2.0 Gy relative biological effectiveness (RBE) for liver metastasis and lymph node metastases, respectively. The liver metastasis and lymph node metastases received total doses of 79.2 and 60 Gy RBE, respectively (Fig. 3).

**Fig. 4:**
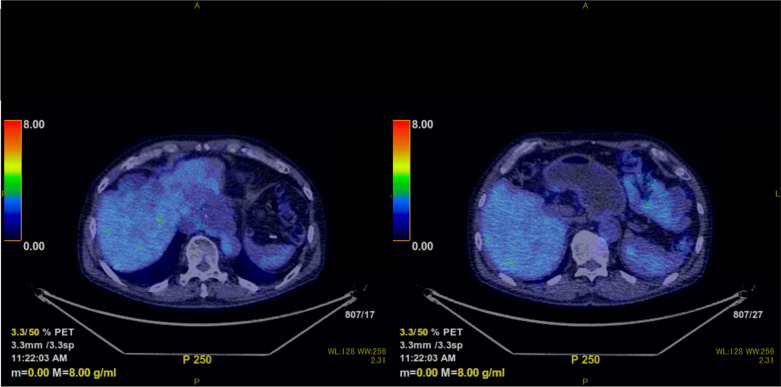
Positron emission tomography after proton beam therapy. Proton beam therapy resulted in the disappearance of fluorodeoxyglucose in all targets

**Fig. 5:**
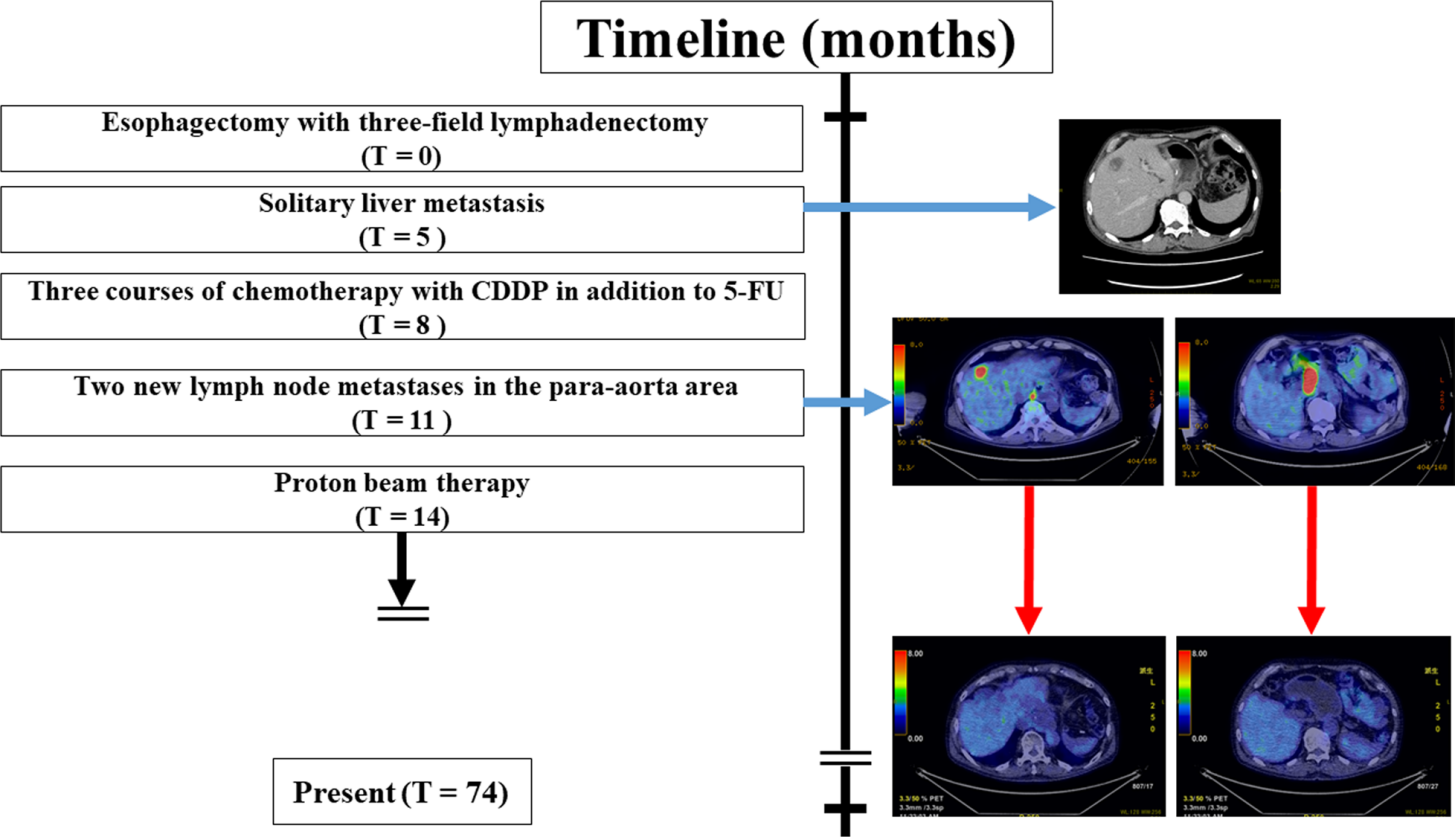
The timeline for intervention and clinical outcome is presented

An acute side effect of grade 1 dermatitis (according to the National Cancer Institute Common Terminology Criteria for Adverse Events version 4.0) occurred after PBT, but there was no acute or late complication of more than grade 2. PET after treatment showed no evidence of recurrence (Fig. 4). The patient remains in complete remission 5 years after treatment without surgery or chemotherapy. The timeline for the present case is shown in Fig. 5.

## Discussion and conclusions

A total irradiation dose of 60–70 Gy has been used for radiation therapy for recurrent ESCC without chemotherapy, and the 5-year overall survival rate was reported to be 11–22% [11]. Murori *et al*. also reported that primary tumors including mediastinal lymph nodes and liver metastases received proton beam irradiation at a total dose of 66–68 Gy RBE with chemotherapy [12]. In addition, it was reported that high-dose (72.0–90.8 Gy RBE) proton beam therapy with or without chemotherapy was an efficacious and safe treatment for patients with ESCC [13].

The present case was treated with PBT for multiple recurrence of ESCC without chemotherapy. It is not surprising that abdominal lymph node metastases from various cancers are well controlled by PBT without chemotherapy; however, there have been few reports on successful treatment with PBT without chemotherapy for distant metastases from ESCC.

Chemotherapy with CDDP and 5-FU is the first-line treatment for unresectable advanced cancer and recurrent ESCC. However, second-line treatment after failure of chemotherapy with CDDP and 5-FU is controversial. Treatment with a single agent such as paclitaxel, docetaxel, navelvine, erlotinib, or irinotecan and some combination therapies have been performed. Previous studies showed response rates of 12.5–44.2% and median survival periods of 6–10.4 months. On the other hand, the median survival period of patients with ESCC for whom first-line therapy failed is only 3–6 months if they do not receive chemotherapy [14–19]. Despite the fact that first-line therapy in our patient with ESCC failed, we were able to control liver metastasis and lymph node metastases due to the high dose of PBT and the biological effective advantage of PBT compared with conventional X-ray therapy.

It has been reported that some patients with malignancies may refuse standard treatment. According to a retrospective review of the Surveillance Epidemiology and End Results (SEER) database between 2004 and 2013, the rate of surgery refusal was 0.64%. It was also suggested in that review that age, ethnicity, marital status, disease stage, and lack of insurance are associated with a higher risk of surgery refusal [10]. Considering this report, it is notable that our unique point was that we were able to use PBT for the patient despite the fact that the patient had refused standard therapy.

Cancer status with less than five metastatic or recurrent lesions and with controlled primary lesions can be considered as “oligorecurrence” [20]. A previous study showed that oligorecurrence in lymph nodes of patients with esophageal cancer can be cured by chemoradiotherapy [21]. Little is known about the survival of patients with specific subtypes of recurrent disease after radical esophagectomy. Patients with a small number of lymph node metastases and patients with metastasis in a solid organ such as the liver, brain, or lung from esophageal cancer have been reported to have a significantly longer median survival period [21, 22]. Therefore, the prognosis may not be poor, even if a small number of lymph node metastases are combined with isolated liver metastases. However, there has been no study on the outcome of treatment when a small number of lymph node metastases in a single solid organ occur at the same time. Generally, patients with organ metastases and lymph node metastases have a poor prognosis. Kato *et al*. reported a classification of recurrent ESCC after radical esophagectomy, with a longer survival period in cases of locoregional recurrence than in cases of distant or mixed recurrence and a shorter survival period in cases of locoregional recurrence in more than one lymph node station and in a solid organ such as the bone, pleura, or peritoneum [22].

The abscopal effect is probably associated with our good local control without chemotherapy for recurrence of ESCC by PBT. This effect is known as an immune effect caused by high doses of radiation and other factors. The biological mechanism underlying this effect remains unclear. There have been few case reports on an abscopal effect for esophageal cancer. Zhao X *et al*. reported an abscopal effect of radiation on lymph node metastasis in an ESCC patient who received CyberKnife radiotherapy with a dose of 42 Gy in six daily fractions [23]. To the best of our knowledge, there has been no study on an abscopal effect of PBT for ESCC.

This is an interesting case report on management of a single liver metastasis with lymph nodal disease progression in the paraaortic area. It is reasonable to consider that our case was oligorecurrence of a solitary organ and localized lymph node metastases with good prognosis. Because this study was a case study, it is difficult to define the indication for salvage therapy for postoperative recurrence of esophageal cancer. However, it is possible that some patients with single solid organ metastasis with localized lymph node metastases were treated only by chemotherapy despite being potential candidates of PBT. Additional research would be necessary for understanding the biological basis on which some patients could enjoy long-term disease control.

PBT for recurrent ESCC and metastatic lesions is considered to be a therapeutic option. We hope that multicenter trials will be carried out and that indications will be expanded.

## Data Availability

The data include individual patient data, but the data are available from the corresponding authors upon reasonable request.

## Abbreviations

ESCC: Esophageal squamous cell carcinoma
CDDP: Cisplatin
CT: Computed tomography
CTV: Clinical target volume
GTV: Gross tumor volume
Gy: Gray
PBT: Proton beam therapy
PET: Positron emission tomography
PTV: Planning target volume
RBE: Relative biological effectiveness
